# Simulation of FES on the forearm with muscle-specific activation resolution

**DOI:** 10.3389/fbioe.2024.1384617

**Published:** 2024-06-27

**Authors:** Johanna Baier, Sascha Selkmann, Beate Bender

**Affiliations:** Chair for Product Development, Institute for Product and Service Engineering, Ruhr-University Bochum, Bochum, Germany

**Keywords:** electromagnetic simulation, upper limb, digital twin, individual model, selective muscle activation, product development, electrical stimulation

## Abstract

**Introduction:**

Functional electrical stimulation (FES) is an established method of supporting neurological rehabilitation. However, particularly on the forearm, it still cannot elicit selective muscle activations that form the basis of complex hand movements. Current research approaches in the context of selective muscle activation often attempt to enable targeted stimulation by increasing the number of electrodes and combining them in electrode arrays. In order to determine the best stimulation positions and settings, manual or semi-automated algorithms are used. This approach is limited due to experimental limitations. The supportive use of simulation studies is well-established, but existing simulation models are not suitable for analyses of selective muscle activation due to missing or arbitrarily arranged innervation zones.

**Methods:**

This study introduces a new modeling method to design a person-specific digital twin that enables the prediction of muscle activations during FES on the forearm. The designed individual model consists of three parts: an anatomically based 3D volume conductor, a muscle-specific nerve fiber arrangement in various regions of interest (ROIs), and a standard nerve model. All processes were embedded in scripts or macros to enable automated changes to the model and the simulation setup.

**Results:**

The experimental evaluation of simulated strength–duration diagrams showed good coincidence. The relative differences of the simulated amplitudes to the mean amplitude of the four experiments were in the same range as the inter-experimental differences, with mean values between 0.005 and 0.045. Based on these results, muscle-specific activation thresholds were determined and integrated into the simulation process. With this modification, simulated force-intensity curves showed good agreement with additionally measured curves.

**Discussion:**

The results show that the model is suitable for simulating realistic muscle-specific activations. Since complex hand movements are physiologically composed of individual, selective muscle activations, it can be assumed that the model is also suitable for simulating these movements. Therefore, this study presents a new and very promising approach for developing new applications and products in the context of the rehabilitation of sensorimotor disorders.

## 1 Introduction

Functional electrical stimulation (FES) has received significant attention in recent years for its positive impact on motor recovery in post-stroke patients or those with spinal cord injury. By applying electrodes to the skin of the forearm, FES can elicit hand movements that otherwise might be impossible. However, achieving precise and physiological hand movements is still challenging (Westerveld et al., 2012; Malesevic et al., 2012; Imatz-Ojanguren et al., 2016); common applications are therefore limited to simple movements such as hand opening and closing. In order to stimulate complex and functional hand movements, different wrist and finger muscles must be selectively activated in a specific pattern (Imatz-Ojanguren et al., 2016). This is particularly difficult at the forearm due to the anatomy—numerous small muscles arranged in several layers (Westerveld et al., 2012; Bao et al., 2018; Baker et al., 1993). A muscle is activated selectively when it is activated while minimizing the activation of neighboring muscles. In addition to the stimulation parameters (current amplitude, pulse width and shape, and frequency) and chosen electrodes (size and geometry), the selective activation of a muscle depends particularly on the stimulation site (Westerveld et al., 2012; Bao et al., 2018; Baker et al., 1993). The best position to stimulate a single muscle is generally referred to as the “motor point” (MP) (Baker et al., 1993; Imatz-Ojanguren et al., 2016; RaviChandran et al., 2020b).

Current research approaches in the context of selective muscle activation often attempt to enable selective stimulation at different MPs by increasing the number of electrodes. Arrays with many small electrodes are placed on the forearm in order to determine the best stimulation positions and settings using manual or semi-automated algorithms (Koutsou et al., 2016; Salchow et al., 2016a; Crema et al., 2018). The evaluation of the stimulated movement is performed either manually by an expert (Salchow et al., 2016a) or automatically by measuring movements (Malesevic et al., 2012; Bao et al., 2018; Salchow et al., 2016b) or forces (Keller et al., 2007). All previous studies use non-specific arrays (electrodes arranged in regular grids) and are based on a limited number of experiments because these are very time-consuming and are further limited by premature muscle fatigue during FES. The search algorithms are therefore not optimized—they require many repetitions and either take a long time or only cover a small range of the possible settings. Despite extensive research, these approaches have not yet succeeded in developing a system suitable for everyday use that reliably and without great additional effort enables the stimulation of selective muscle activation in the forearm as a basis for complex hand movements. It is thus evident that other or complementary methods are needed. Particularly in the field of biomedical engineering, there is great potential in coupling human-related models with digital twins using biomechanical or bioelectronic simulations (Neumann et al., 2020).

In the context of FES, simulation studies have been successfully used to enhance understanding and support the development of new FES applications (Imatz-Ojanguren et al., 2016). Existing simulation studies have primarily focused on two influencing factors: electrode configuration and fat-layer thickness. Evaluation criteria in simulation-based analyses often cover either the activation of single modeled nerve fibers or a general volume area in which fiber activation occurs (activation volume). For instance, Gomez-Tames et al. (2011) analyzed the influence of fat-layer thickness on activation volume, and five other studies have investigated the interdependency of fat thickness and electrode size (Doheny et al., 2010; Gomez-Tames et al., 2012; Gomez-Tames et al., 2013b; Kuhn and Keller, 2005; Kuhn et al., 2010), inter-electrode distance (Doheny et al., 2010; Gomez-Tames et al., 2012; 2013b), and electrode shape (Gomez-Tames et al., 2012). With respect to the electrode configuration, Kuhn et al. (2009b) analyzed the influence of electrode material properties (hydrogel resistivity) and composed electrodes (as often used with array electrodes) on the required stimulation current to achieve a defined activation (depth of activation volume). Cooper et al. (2011) also addressed electrode material properties and simulated resistivity changes during prolonged use. Two other studies have addressed just the electrode shape with respect to the nerve fiber orientation: Goffredo et al. (2014) analyzed different patterns of an electrode array; Loitz et al. (2015) rotated a rectangular single electrode. In contrast, RaviChandran et al. (2020a) analyzed the influence of electrode shape in terms of different edge length but same area. However, none of the studies analyzed the influence of stimulation settings and person-specific characteristics on the selective activation of individual muscles, taking into account the surrounding muscles. Such studies are critical to support experimental improvement and enable the transfer of these approaches to daily practice.

Existing simulation models only represent the “outer” anatomy (i.e., the different homogeneous tissue layers) and are not suitable for analyses for selective muscle activation due to missing or randomly arranged innervation areas. All models cover a 3D volume conductor with one or various embedded nerve fibers which are coupled to a nerve model. This two-step approach was first introduced by McNeal (1976). The volume conductor is used to simulate the extracellular potential distribution at the nerve fibers. On this basis, the activation of the nerve fibers is then calculated with the nerve model. All existing 3D volume conductors consist of three to five homogeneous tissue layers, mostly arranged in symmetrical cylinders. In contrast, past models differed in the arrangement of the nerve fibers: single nerve fibers at specified locations (Doheny et al., 2010; Cooper et al., 2011; Gomez-Tames et al., 2013b; RaviChandran et al., 2020a), nerve fiber bundles at specified locations (Goffredo et al., 2014; Kuhn et al., 2009b; 2010), many nerve fibers homogeneously distributed in the whole muscle layer (Kuhn and Keller, 2007), and many nerve fibers located in a specified volume representing one single muscle (Gomez-Tames et al., 2013c; a; Loitz et al., 2015). However, none of the existing models provides muscle-specific activation resolution, which is required to use the simulation to develop new applications and products that enable the stimulation of complex hand movements.

This paper introduces the design and evaluation of a new, person-specific simulation model that enables the analysis and evaluation of simulation results at the level of selective muscle activations. In contrast to previous models, our volume conductor is anatomy-based, which means that an individual anatomy given by MRI data is approximated by regular geometries. Furthermore, the nerve fibers in our model are arranged in various muscle-specific regions. This allows conclusions to be drawn about the activation of each individual muscle as the result of a stimulation pulse at a specific electrode position. The developed model is evaluated with experimental measurements and shows good alignment for strength–duration and force-intensity curves. Thus, the new model enables the simulation of single muscle activation, which forms the basis for complex movements and further simulation-based analysis.

The two main objectives of this paper are as follows:

• The introduction of a new modeling method to develop a digital twin of the forearm that accurately and reliably predicts real stimulated muscle activations for different electrode configurations and stimulation settings.
• The automation of model modifications and simulation workflow in order to enable the future use of the digital twin in product development with respect to optimization and AI methods.

## 2 Methods

**FIGURE 1:**
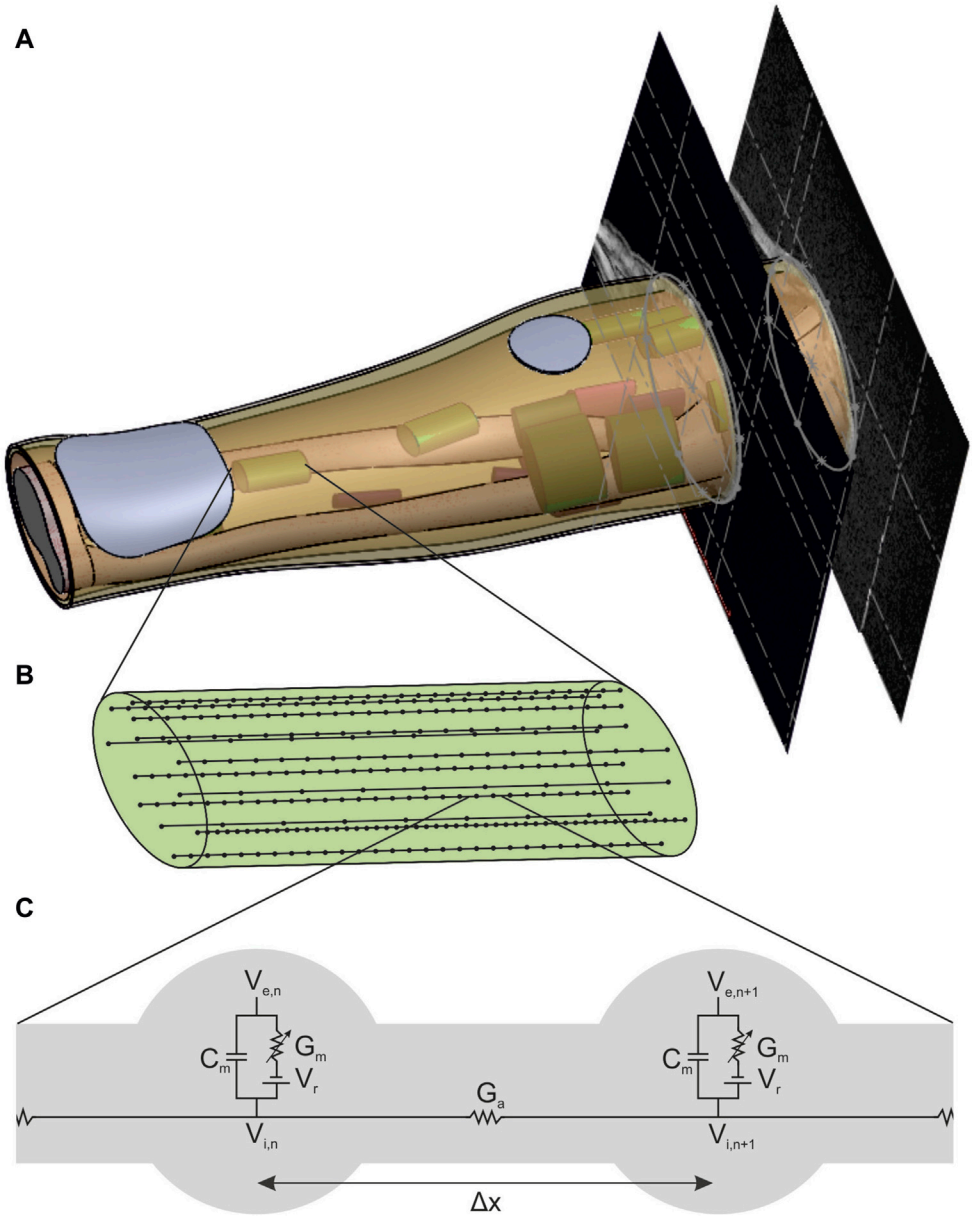
New forearm model enabling the simulation of FES with muscle-specific activation resolution. **(A)** Anatomically based volume conductor with four homogeneous tissue layers (bone, muscle, fat, and skin) and two stimulation electrodes that is extended with muscle-specific regions of interest (ROIs) to support the nerve fiber arrangement. **(B)** Detailed view of one ROI with 15 exemplary nerve fibers with random positions and random fiber diameter based on a bimodal distribution. **(C)** Detailed view of the connection of two nodes of ranvier of a nerve fiber with the linear cable model based on McNeal (1976).

The new individual model basically consists of three parts: an anatomically based 3D volume conductor with four homogeneous tissue layers and two stimulation electrodes placed on the skin surface (Figure 1A), a muscle-specific nerve fiber arrangement in various regions of interest (ROIs) (Figure 1A, B), and a standard nerve model connected to the single nerve fibers (Figure 1C). According to the two-step approach mentioned above, the volume conductor was used to simulate the extracellular potential distribution caused by a single stimulation pulse, and the nerve model was used to simulate the activation state of the single fibers based on this potential distribution. In addition, individual muscle activations are calculated based on the simulated fiber activations, which is enabled by the muscle-specific arrangement of nerve fibers. This model and simulation setup is then evaluated by comparing simulated strength–duration (SD) diagrams with experimental measurements.

### 2.1 Volume conductor and potential distribution

The volume conductor consists of four anatomically based homogeneous tissue layers: a bone layer covering the two forearm ulna and radius bones as well as the elbow joint surrounded by muscle tissue and a fat and a skin layer (Figure 1A). In addition, two electrodes consisting of a hydrogel pad and an electrode sheet on the top are modeled on the skin layer. It is based on the MRI data of a healthy subject (34y, female, position of the forearm during scan: elbow flexed 90°, forearm parallel to shoulders, hand in pronation position). The subject gave her informed consent to the use of the MRI data for modeling and to the publication of the resulting model. No personal data are included, and no conclusions can be drawn. The MRI data were used exclusively as an anatomical reference for modeling; no medial application took place. Therefore, no ethical approval was required for data collection and their use for modeling.

For the design, the contours of the two bones, the muscle–fat interface, and the skin were approximated by single or composed (only at the elbow bone) ellipses in 10 MR images: 8 MR images equidistant between the lateral epicondyle of the humerus (LEH) and the radial styloid process (RSP), one additional image at the proximal end of the radius, and another 5% of the LE-RSP distance proximal to the LEH. This approach, using regular geometries to approximate the anatomic shape, ensured that the resulting volumes and surfaces were still mathematically defined and therefore that the model can be easily extended or modified. The chosen approach also implied the use of 3D design software (Solidworks, Version 2018; Dassault Systems; Velizy-Villacoublay, France) instead of, for example, an MRI viewer with rendering options. The space between LEH and RSP is the main part of the model due to the reference system for the ROI positioning (see section 2.2) and has a length of 242 mm; the proximal extension is then required to cover the most proximal ROI entirely and leads to a total model length of 254.1 mm. Five filled volumes were created using the molding feature that combines the contours of each set: radius and ulna (only until LEH), elbow bone (from LEH to end), and muscle and skin (whole length). The three bone volumes were then combined into a single volume (bone layer). In addition to the filled volume, a thin molding feature 1 mm thick was constructed from the skin contours (skin layer). The other layers were then constructed by subtraction operations: filled muscle volume minus bone volume (muscle layer) and filled skin volume minus filled muscle volume and skin layer (fat layer).

To complete the 3D volume conductor, the two electrodes were designed on the skin so that they could be modified in position, geometry, and size. In this step, changes in electrode configuration were processed automatically by incorporating a macro and a linked text-file. The position of each electrode is defined by an axial and a radial value. The axial position is the distance of the electrode along the forearm from the LEH; the radial position is the distance of the electrode from a predefined reference line along the circumference counterclockwise (looking from elbow to wrist) of the skin at that axial position. The reference line is a straight line that divides the ulna approximately in the middle. It is constructed as the intersection between a plane positioned manually at the right height and the skin surface. To construct each of the electrodes, the following steps were performed:

1) The electrode position is constructed: First, a plane parallel to the top face of the arm at the distance of the axial position was added and an intersection curve of the skin at that position was sketched. This curve was then trimmed at the end (counterclockwise) by adding a short straight line (0.5 mm). Finally, the electrode position was added by a 3D reference point that was placed on the open intersection curve at the percentage distance corresponding to the radial position.

2) The electrode geometry is constructed: The geometry was sketched, including all dimensions on a plane tangential to the skin at the constructed electrode position. To allow a full projection of that sketch onto the skin surface, a plane with 20 mm offset from the model was used.

3) The hydrogel volume is constructed: The skin layer was first copied twice. The electrode sketch was then wrapped around the outer surface of the skin copy (specifications: Spline Surface and Emboss with 1 mm thickness). The second skin copy was then subtracted from the first to obtain the hydrogel volume without the skin layer.

4) The electrode face is constructed: The outer surface of the hydrogel volume was copied. In the case of array electrodes, various surfaces were added through additional planes and sketches (not used in this study).

The resulting volume conductor, consisting of four anatomically based homogeneous tissue layers and two hydrogel electrodes, was then used to simulate the extracellular potential using the finite element method (FEM). Thereby, the simulated stimulation impulse was transmitted through the modeled electrodes. Since the propagation of the stimulation pulse in biological tissues is an electromagnetic field problem, it can be described by Maxwell’s equation. By assuming quasi-stationarity (Plonsey and Heppner, 1967) and neglecting the magnetic flux, which is not of interest in the present case, Maxwell’s equation simplifies thus:

$$-\nabla \cdot \left(\left[\epsilon \right]\nabla \frac{\partial V}{\partial t}\right)-\nabla \cdot \left(\left[\sigma \right]\nabla V\right)=0.$$ (1)

Standard simulation software uses this equation, such as Ansys Electronics Desktop (2022 R2; Ansys Inc.; Canonsburg, Pennsylvania, United States) in the Maxwell package used in this study. To set up the simulation, the material properties are first defined, and then meshing is followed by the stimulation pulse.

The connection between the electrodes and skin was modeled by a simplified electrical network based on resistive and capacitive elements. This modeling approach, proposed by Keller and Kuhn (2008), provides a basis for understanding the interactions at the interface between electrode and skin. However, this approach does not account for inhomogeneities within the tissues and the electrode, such as sweat glands or blood vessels. According to Kuhn (2008), these inhomogeneities only have a significant impact on nerve activation when they are in close proximity to the nerves. For the purposes of this study, which aims to analyze nerve activation solely within the muscle volume, this simplified model proved sufficient, particularly as the anisotropic material properties of the muscle and the isotropic properties of the other tissues were considered.

The material properties cover the biological tissues of the forearm as well as the hydrogel of the electrodes. Electrical properties of biological tissue include conductivity and permittivity due to their microanatomy with closed compartments covering a liquid embedded in another liquid. Both properties depend on the exact tissue composition and structure (e.g., water content), and the experimentally determined values in the literature are inconsistent. In the context of the simulation of transcutaneous stimulation, Kuhn and Keller (2005) compared simulated surface and intramuscular potential curves of six models with different material properties with experimentally measured curves. Based on their results, the authors defined standard materials that were used in this study (Table 1), as in several previous FES simulation studies.

**TABLE 1:** Material properties (conductivity *σ* and relative permittivity *ϵ*_*r*_) defined by Kuhn and Keller (2005) as standard materials and meshing operations for hydrogel of the electrodes and biological tissues.

Layer	Material properties		Mesh operations	
	Conductivity *σ* [*S*/*m*]	Relative permittivity *ϵ*_*r*_ [-]	Type	Maximum length [*mm*]
Hydrogel^a^	1/11	1	inside	2
Skin	1/700	6,000	inside	2
Fat	1/33	25,000	inside	4
Muscle (axial)	1/3	120,000	inside	7.5
Muscle (radial)	1/9	40,000		
Bone^b^	1/50	3,000	inside	10

^a^ Material properties modified according to the product data sheet.

^b^ Material properties from cortical bone used for the whole bone layer.

The meshing was realized in Ansys Electronics Desktop using tetrahedral elements and refining them by manual mesh operations for each layer. Table 1 summarizes the mesh operations and parameters used, which were selected on the basis of a convergence analysis.

The stimulation pulse was modeled by a current placed on a different electrode surface near the elbow and a sink placed on the indifferent electrode surface at the wrist. To obtain a single rectangular pulse with ramp-in and -out as used by the stimulator, a piece-wise-linear function (pwl) was used to define the current. Furthermore, Ansys autonomously selected the boundary conditions for the electrodes based on the type of excitation. The external environment (air) was neglected in solving the problem.

### 2.2 Muscle-specific nerve fiber arrangement

The muscle-specific arrangement of nerve fibers was realized by grouping them into distinct regions of interest (ROIs), with each ROI approximating the innervation zone (IZ) of a muscle at the nerve entry point (NEP) of the corresponding muscle. This is realized by determining first the NEP of a muscle to define the axial position of the ROI, then approximating the muscle course to define the orientation, and thirdly approximating the cross section of the muscle by an ellipse to define the size. The NEPs were determined by mapping data from studies on cadaveric human forearms to the anatomy on which this work was based (given by the MRI data). Table 2 summarizes the proximal maximum, median, and distal maximum distance of the NEPs from the lateral epicondyle of the humerus (LEH) relative to the forearm length (defined as the distance between the LEH and the radial styloid process, or RSP) for all forearm muscles. Most values are based on Liu et al. (1997). They analyzed the innervation pattern (number and position of NEPs) in ten cadaveric forearms. Their results showed that the NEPs of the two finger flexors vary along more than 50% of the forearm, so additional data were taken into account to split these ranges in two ROIs (Bickerton et al., 1997; Hwang et al., 2007). Negative values were excluded for modeling as the authors examined extended arms in contrast to the MRI data used that cover a flexed arm: the BR was not included at all and the extensor carpi radialis longus (ECRL) was designed without using the proximal maximum value. A total of 20 muscle-specific ROIs were added to the 3D forearm model by the following steps.

1) The axial distances given in Table 2 were calculated, and the nearest MR image was determined and added at the corresponding distance to the existing 3D forearm model. The median section defined the axial position of the ROI.
2) The required muscle was identified in all three MRI images. In each slice, an ellipse approximating the cross section of the required muscle was designed, where the three major axes had to be parallel. To support this step, a continuous 3D segmentation of all muscles was realized using a 3D Slicer (http://www.slicer.org). In this segmentation, the dimensions and the angle (roll) of the single ellipses were determined. The orientation of the ROI was defined then by first connecting the major axes (pitch) on the sections at the proximal and distal maximum and then the minor axes (yaw).
3) The area of the ROI was sketched on a tilted plane (at the defined axial position, perpendicular to the defined orientation) and extended 10 mm proximally and distally. Each ROI has an elliptical area with muscle-specific dimensions.

**TABLE 2:** Overview of the literature values used to position the ROIs at the supposed nerve entry points (NEPs). The main study by Liu et al. (1997) examined several cadaveric forearms to determine the number and position of terminal nerve branches entering a muscle.

Muscle group	Muscle name		Proximal Max^a^ [%]	Median^a^ [%]	Distal Max^a^ [%]
Superficial flexors	Flexor carpi radialis	FCR	16	25	37
	Flexor carpi ulnaris	FCU	6	13	51
	Flexor digitorum superficialis	FDSdist	(−5.5)	74^b^	(+5.5)
		FDSprox	(−5.5)	51.5^b^	(+5.5)
	Palmaris longus	PL	10	18	35
	Pronator teres	PT	5	16	28
Deep flexors	Flexor digitorum profundus	FDPdist	(−5.5)	36.7^c^	(+5.5)
		FDPprox	(−5.5)	26.5^c^	(+5.5)
	Flexor pollicis longus	FPL	25	40	60
	Pronator quadratus	PQ	72	85	90
Radial extensors	Brachioradialis	BR	−17	−4	12
	Extensor carpi radialis brevis	ECRB	12	25	37
	Extensor carpi radialis longus	ECRL	−8	3	15
Superficial extensors	Extensor carpi ulnaris	ECU	25	33	43
	Extensor digitorum	ED	17	33	52
	Extensor digiti minimi	EDM	34	37	60
Deep extensors	Abductor pollicis longus	APL	34	40	52
	Extensor indicis	EI	56	65	87
	Extensor pollicis brevis	EPB	41	61	70
	Extensor pollicis longus	EPL	39	52	67
	Supinator	SUP	10	19	31

^a^ (Liu et al., 1997).

^b^ (Bickerton et al., 1997).

^c^ (Hwang et al., 2007).

**FIGURE 2:**
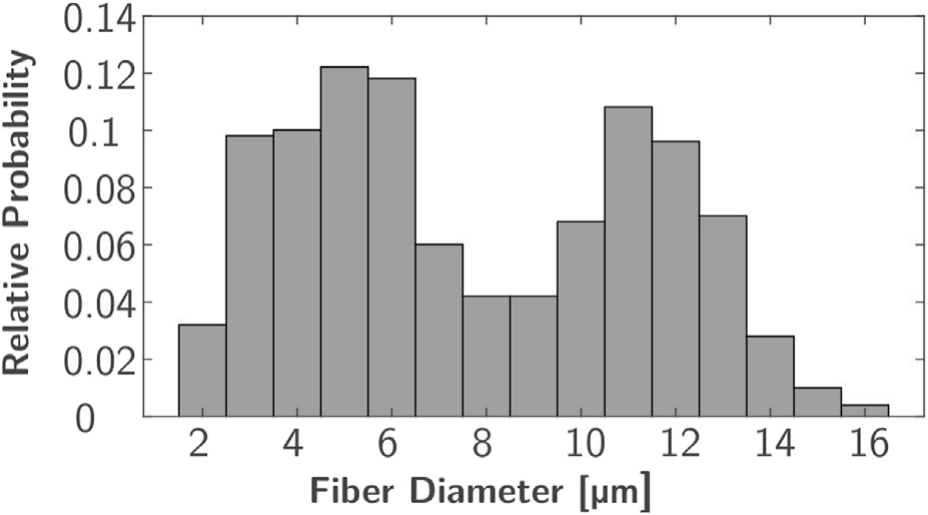
Bimodal diameter distribution used for nerve fiber modeling based on averaged distributions from Buchthal and Rosenfalck (1966) and O’Sullivan and Swallow (1968).

In each ROI, 500 uniformly distributed nerve fibers underlying a bimodal diameter distribution were modeled. Every nerve incorporates various fiber types that can be distinguished by diameter and differ in their characteristics, such as conduction velocity and excitability (Rattay, 1990; Hall and Guyton, 2011). The bimodal diameter distribution has been determined for myelinated fibers in peripheral nerves in animals (Prodanov and Feirabend, 2007; Rijkhoff et al., 1996) and humans (Buchthal and Rosenfalck, 1966; O’Sullivan and Swallow, 1968; Jacobs and Love, 1985). In this study, diameter distributions of two median nerves (Buchthal and Rosenfalck, 1966) and a sural nerve (O’Sullivan and Swallow, 1968) (which is similar to the distributions of the analyzed radial nerves) were averaged. The resulting distribution ranges from 2 to 16 µm and has peaks at 5 and 11 µm (Figure 2); it is comparable to the fiber distributions used in previous FES simulation studies (Kuhn et al., 2009a; Gomez-Tames et al., 2013a; Loitz et al., 2015). As no 3D nerve model was incorporated but each fiber was represented by a single line with nodes at fiber-specific distances (Figure 1C, Δ*x*), this modeling was realized in MATLAB (Version R2024a; The MathWorks, Inc.; Natick, Massachusetts, United States). To align the positions with the modeled ROIs, these were exported to MATLAB as stl-files. The modeled nodes represent the nodes of Ranvier between the myelin sheaths where the electrophysiological processes occurr in saltatory conduction (Hall and Guyton, 2011), and their distance increased with fiber diameter. The chosen Δ*x* values were based on McIntyre et al. (2002) and the first and last nodes were of random distance to the ROI bottom and top faces.

### 2.3 Nerve model and muscle activation

This study used two common approaches to predict the effect of external stimulation on the nerve fibers: the linear cable model (LC) and the activating function (AF) (McNeal, 1976; Rattay, 1986; 1990). Both approaches were chosen due to their computational efficiency and have already been successfully used in the context of optimization problems (Loitz et al., 2015) and implemented in MATLAB. The linear cable model is the simplest electrical representation of a nerve fiber, first introduced in McNeal (1976). It combines all ionic currents at the Ranvier nodes into one time-invariant current and it assumes the myelin sheath to be a perfect insulator so that the internode section can be modeled by a stand-alone conductance. Eq. (2) gives the resultant mathematical description for node *n* (Figure 1C, left node).

$$\frac{dV_{m,n}}{dt}=\frac{1}{C_{m}}\left[G_{a}\left(V_{m,n-1}-2V_{m,n}+V_{m,n+1}+V_{e,n-1}-2V_{e,n}+V_{e,n+1}\right)-I_{\mathrm{ionic}}\right].$$ (2)

If the calculated membrane potential exceeds a defined threshold, the corresponding node of Ranvier is assumed to generate an action potential (McNeal, 1976) and the nerve fiber is considered activated (Kuhn, 2008; Loitz, 2019). Table 3 summarizes all axon and membrane characteristics used to build the linear cable model in MATLAB.

**TABLE 3:** Overview of the axon and membrane characteristics used to set up the linear cable model.

Parameter		Value	References
Axon diameter	*d*	2–16 μm	Buchthal and Rosenfalck (1966); O’Sullivan and Swallow (1968)
Internodal distance	Δ*x*	155–1,500 μm	McIntyre and Grill (1998)
Node length	*L*	2.5 μm	McNeal (1976)
Specific axon resistance	*ρ*_*i*_	0.7 Ω*m*	McNeal (1976)
Membrane conductance/unit area	*g*_*m*_	30.4 *m*/*cm* ^2^	McNeal (1976)
Membrane capacitance/unit area	*c*_*m*_	2 *μF*/*cm* ^2^	McNeal (1976)
Resting potential	*V*_*r*_	−70 *mV*	McNeal (1976); Frankenhaeser and Huxley (1964)
Threshold potential	*Vth*	−55 *mV*	

The AF is a simplified mathematical description for predicting the nerve fiber response defined as the second derivative of the extracellular potential along the axon course. Rattay (1986) initially showed that this is the main driver for the generation of action potentials in unmyelinated axons.

$$f\left(x,t\right)=\frac{\partial^{2}V_{e}\left(x,t\right)}{\partial x^{2}}.$$ (3)

A positive value indicates membrane depolarization and a negative value, hyperpolarization (Rattay, 1986; 1999). Although this term was initially defined for unmyelinated axons, it has also been successfully used to predict the activation of myelinated fibers during FES simulation (Kuhn, 2008; Cooper et al., 2011; Gomez-Tames et al., 2011; 2013b; Loitz, 2019). Kuhn (2008) further introduced thresholds depending on the most important influencing factors (axon diameter and pulse width) to improve prediction accuracy when simulating FES with skin electrodes. These thresholds were used in the current study when predicting fiber activation with the activating function (for more details and the lookup values, see Kuhn, 2008).

Based on the single fiber activations, the percentage of activated fibers within one ROI was calculated, which is proportional to the muscle activation as outlined above. This was done for every ROI so that a muscle-specific activation resolution was achieved.

### 2.4 Experimental evaluation

The experimental evaluation was realized by comparing simulated and experimentally measured strength–duration (SD) diagrams for eight ROIs at predefined electrode positions. SD diagrams relate the required amplitude to achieve a defined motor response (mostly motor threshold) to the duration of the stimulation pulse (pulse width) for a specific muscle (Baker et al., 1993). They are a common method for evaluating the excitability of nerves or muscles in experiments (e.g., Baker et al., 1993; Imatz-Ojanguren et al., 2016) and have been used in previous studies to evaluate FES simulation setups (e.g., Kuhn et al., 2009a; Goffredo et al., 2014). The experiments were performed with the healthy subject (34y, female) who also provided the MRI data. Table 4 summarizes the selected settings and used outcome criteria to generate the SD diagrams. At each electrode position, amplitude and pulse width were varied in the specified ranges. The eight ROIs were chosen in a first step because good selective activation can be achieved for these muscles.

**TABLE 4:** Overview of selected muscles, stimulation settings, and outcome criteria to evaluate the new FES simulation model.

	Simulation	Experiment
Muscles	ECU, ED, ECRB, ECRL, FCR, FDS, and FCU	
Amplitude	1–50 mA, increment 1 mA	1 − *x* mA, increment 1 mA, *x* as required to see a motor response
Pulse width	1–500 *μs*, continuous	20–100 *μs* with increment 20 *μs* and 150–500 *μs* with increment 50 *μs*
Motor response	Muscle-specific ROI activation	Significant increase in force

The predefined electrode positions correspond to the projections of the eight ROI centers onto the skin, along with one of the ellipsis axes or between them, depending on the orientation of the ROI. Table 5 summarizes these positions. For the simulations, the positioning is carried out as described above. For the experiments, the same reference system is transferred to the real forearm: a reference line along the ulna (palpable) is drawn on the forearm beginning vertically at the LE of the humerus and ending at the RSP. The axial position is then the length along this line; the radial position is the length along the circumference at height of the axial position. Since the exact dimensions of the model and the real forearm differ due to the model’s simplifications, relative positions are used. To account for the variance in electrode position during the experiments, they were repeated four times. Experiment 1-0 was the initial experiment and marking of the electrode positions. Experiment 1-1 was a replication of 1–0 but included removal and reattachment of the electrodes. Experiments 2-0 and 3-0 were full replications with new marking of the electrode positions. The three new marking procedures resulted in positions within a maximum range of 1 cm.

**TABLE 5:** Overview of selected muscles, settings, and outcome criteria to evaluate the model and simulation setup.

ROI	Different electrode		Indifferent electrode	
	Axial pos. [%]	Radial pos. [%]	Axial pos. [%]	Radial pos. [%]
ECU	33.06	9.88^a^	88.00	20.00
ED	33.06	22.03^a^		
ECRB	25.62	32.11^b^		
ECRL	3.31	30.17^a^		
FCR	25.62	65.36^a^	91.50	72.00
FDSprox	52.07	75.39^b^		
FDSdist	74.38	71.50^b^		
FCU	13.22	85.13^a^		

^a^ Projection in the direction of the minor axis of the ellipse.

^b^ Projection in the direction of the minor and major axes of the ellipses, position in between both points.

**FIGURE 3:**
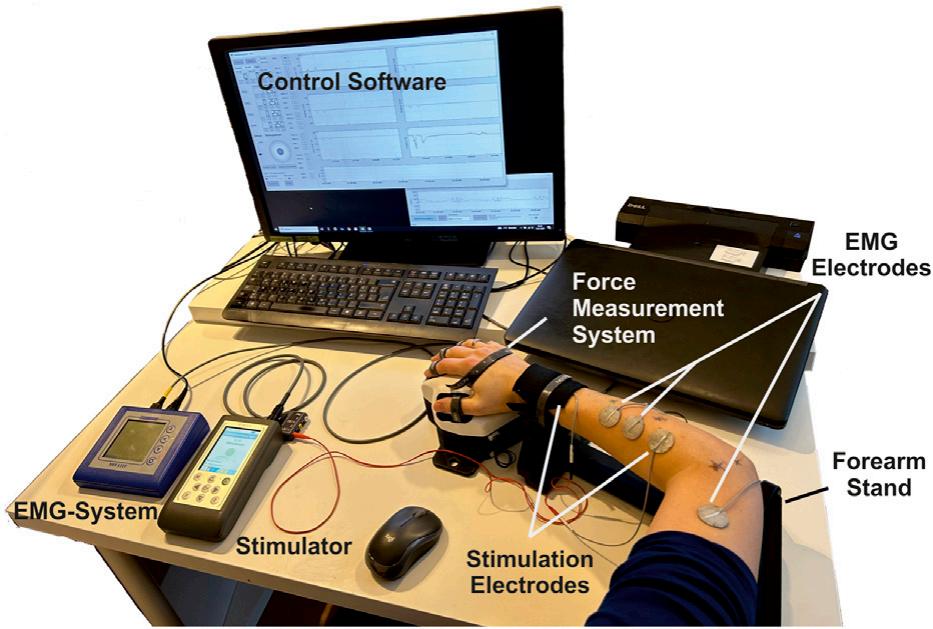
Overview of the experimental setup consisting of a stimulator with two hydrogel stimulation electrodes, a force measuring system with forearm support and control software, and an EMG system with two measuring electrodes and an additional reference electrode.

The experimental setup is shown in Figure 3. It covers the following components and setup.

• A multichannel stimulator (KT Motion, MEDEL GmbH; Hamburg, Germany; CE0483) connected to the skin by two hydrogel electrodes (Axelgaard Manufacturing Co., Ltd.; Fallbrook, CA, United States). The different electrode is round with a diameter of 25 mm and is placed at the stimulation position at the muscle. The indifferent electrode is square with a 50 mm edge length and is placed at a neutral position near the wrist (ventral for flexors, dorsal for extensors).
• A force measurement system was used for the hand and wrist, including an adjustable forearm support and comprehensive software for data recording and stimulation control (self-developed, Chair of Product Development, Ruhr-University Bochum; Bochum, Germany): The hand-rest of the measuring system was ergonomically shaped and allowed placement, in combination with the forearm support, of the hand in a resting position for measurements to avoid forces caused by pre-tensioning of the muscles. The forces generated by isometric muscle contraction were recorded using eight force sensors: two at the base to record the forces resulting from flexion/extension and radial/ulnar abduction of the wrist (±200 N), two for the thumb to record the forces resulting from abduction/adduction (±50 N) and extension/flexion (±200 N), and one for each finger II to V to record the resulting forces from flexion/extension (±50 N). Each force sensor was previously tested for linearity and measurement deviation within the permissible measurement range using a materials-testing machine (Zwick Z010 with GTM GmbH 238 series K, 10 kN, 2 mV/V) and were then calibrated. Both the fingertip and the respective distal joint can be used as the contact point for the force measurements of fingers I to V. The measurement frequency can be set in discrete steps from 10 to 320 Hz. We chose, for our measurements, a frequency of 80 Hz. In addition to the force data, the stimulation settings frequency, pulse width, and stimulation current were recorded in parallel in the software.
• An EMG System (Novativ, MEDEL GmbH; Hamburg, Germany; CE0482) was connected to the skin by three hydrogel electrodes (Axelgaard Manufacturing Co., Ltd.; Fallbrook, CA, United States; EC No 1907/2006): two electrodes for measuring the differential muscle potential between the stimulation electrodes on the respective muscle body and one reference electrode at a neutral position at the upper arm. No exact positioning of the measurement electrodes was carried out, as the influences due to translation along the muscle fibers or rotations up to 30° mainly affect the amplitude of the signal (Duarte et al., 2016), which is not of interest here. The EMG measurements (measurement frequency: 4,500 Hz) were used during the experiments to ensure that no unintended, voluntary muscle activation was performed. A stimulated movement only leads to clearly identifiable M-waves in EMG that occur after each stimulation pulse (Merletti et al., 1992). Any other activation indicated an unintentional voluntary contraction, so the measurement was aborted and repeated.
• A defined hand to forearm to upper arm positioning reflecting the same positioning chosen during MRI data acquisition: hand in pronation position (preset by the measuring system), elbow flexed approximately 90°, upper arm parallel to palm (that is, parallel to the table).

## 3 Results

**FIGURE 4:**
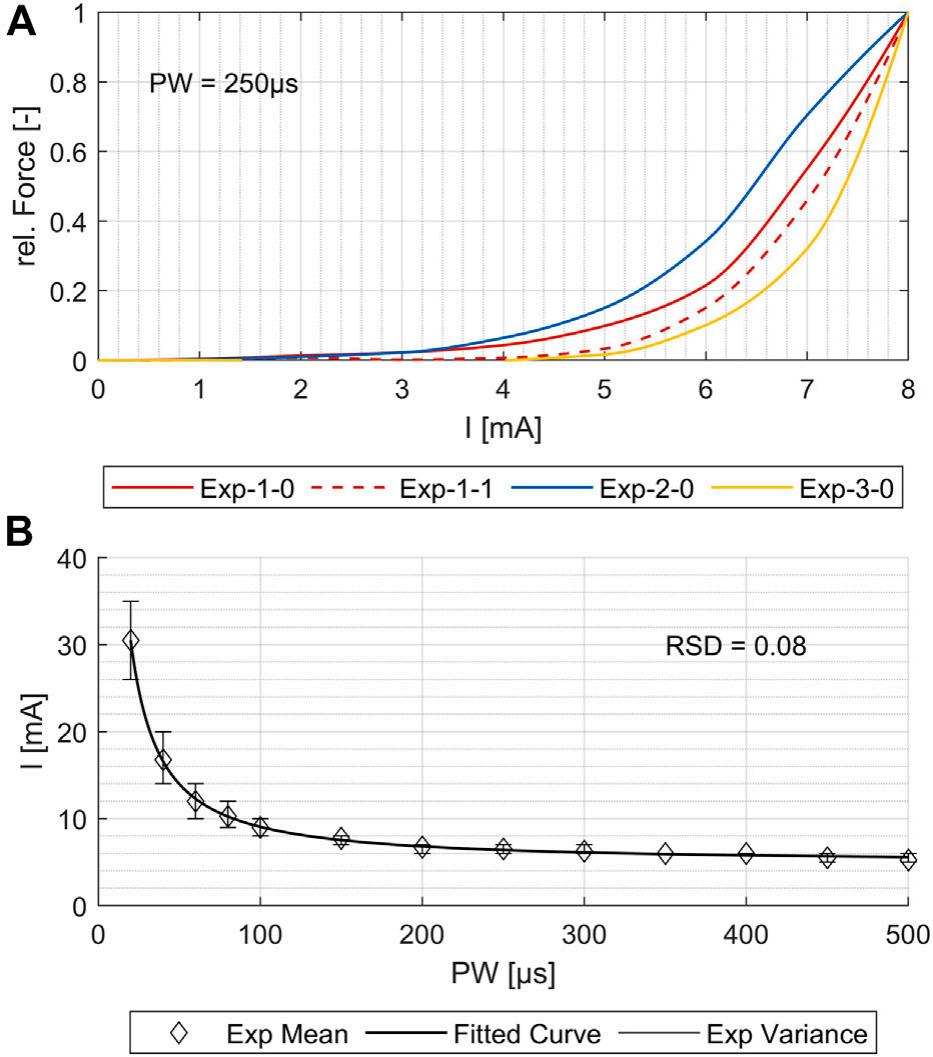
Comparison of the four experimental measurements for one exemplary muscle (flexor carpi radialis, or FCR). **(A)** Force increase over amplitude for PW = 250 μs. **(B)** Variance of the strength–duration diagrams and the resulting fit curve for the mean values. The relative standard deviation (RSD) is 0.08.

Previous models have been limited in simulating the activation of several single muscles, which is necessary for using them as a digital twin and supporting the development of individualized applications and new products. To demonstrate that our simulation model maps real muscle activations correctly and reliably, we generated strength–duration (SD) diagrams for various muscles, both for simulations and the experimental measurements. Typically, in experimental studies, the lowest muscle response is chosen for the creation of the SD diagram. However, in simulations, even at low intensities, individual nerve fibers are activated; such activations may not be measurable in experiments and could lead to an overestimation of activation in the simulation compared to the experiments. To ensure reproducible and comparable thresholds from both experimental measurements and simulations, we opted for the onset of the characteristic sigmoidal progression of the intensity-force curve instead of the usual first muscle twitch. For the example shown in Figure 4A, this means that the activation thresholds used to create the SD diagrams were shifted by force increases from 3–4 mA to 6–7 mA. This applied to all four repetitions of the experiment. Figure 4B shows, for the same muscle, the fitted mean curve that was used for the further comparisons to simulated SD diagrams. Additionally, the maximal variance for the four repetitions is shown. Although the three electrode positions differed (<1 cm) when repeating the marking procedure, the resulting SD plots show only small differences for all muscles with relative standard deviations (RSDs) between 0.04 and 0.09, except for the ECRL, which is slightly higher at 0.14.

**FIGURE 5:**
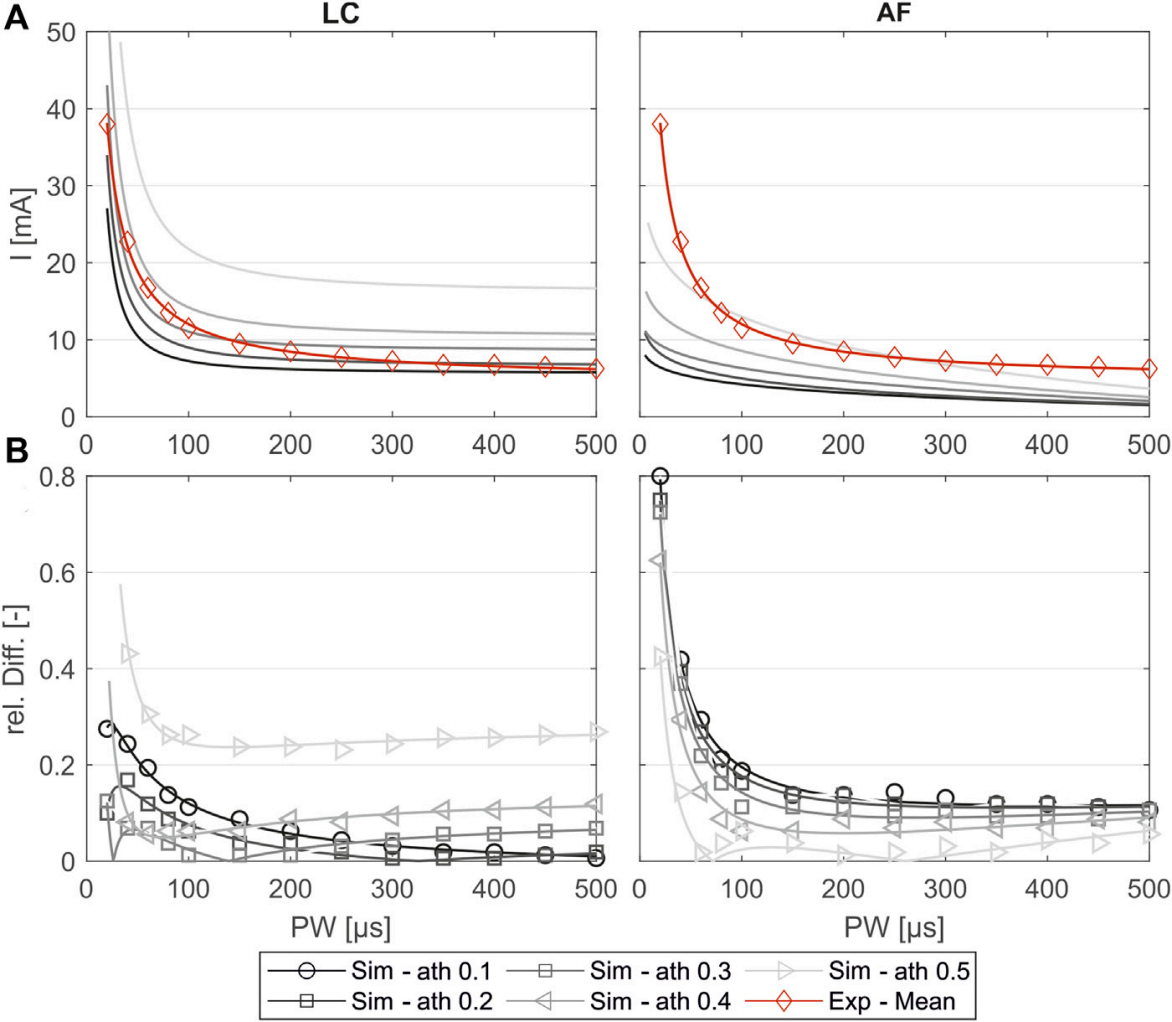
Comparison of the experimentally measured and simulated SD diagrams using the linear cable model (LC) or the activating function (AF) for one exemplary muscle (extensor carpi ulnaris, or ECU). **(A)** Simulated SD curves using different activation thresholds (ath) compared to the mean fit from the experiments. **(B)** Relative difference of the simulated and experimental SD curves: markers show the relative difference at the experimentally measured PW steps, and the lines show the relative difference of the fitted curves shown in **(A)**.

Figure 5A shows the simulated SD curves for one exemplary muscle (extensor carpi ulnaris, or ECU) using the linear cable model (LC) as well as the activating function (AF) compared to the experimental SD curve. The simulated SD curves vary depending on the chosen activation threshold (ath), which is defined as the percentage activation of the corresponding region of interest (ROI). To determine which activation thresholds are appropriate, we conducted the following analysis. Figure 5 includes uniformly distributed ath between 0.1 and 0.6 to illustrate the influence of this factor and the range of the resulting SD curves. In general, the graphs generated with the LC model follow the same trajectory as the experimental curve, whereas the graphs generated using the AF especially fail to map the characteristic curve for low pulse widths (PW) up to 200 µs. Therefore, we excluded the AF in further analysis. To analyze the deviations of the simulated and experimental SD curves, the relative differences were plotted for each PW in 1 µs steps. As illustrated in Figure 5B, the SD graphs for the LC model fit for either low PW or those higher. The curves of the ath with comparable overall good fit intersect at PW 200 ± 50 μs. We therefore determined muscle-specific activation thresholds for two PW ranges, 20–200 μs and 201–500 μs, based on the mean of the relative differences between the SD curves at each PW in 1 μs steps. Table 6 summarizes these values for the different muscles and activation thresholds, with the best fitting cases highlighted in bold. For the muscles that could not be selectively/experimentally validated, we chose the activation thresholds based on the average of the validated muscles for the respective pulse width ranges: 0.20 for *PW* = 20–200 μs and 0.12 for *PW* = 201–500 μs.

**TABLE 6:** Mean relative differences between the simulated and experimental SD curves for two PW intervals: 20 − 200 *μs* and 201 −500* μs*.

ROI	PW range [μs]	Activation threshold (ath)									
		0.05	0.10	0.15	0.20	0.25	0.30	0.35	0.40	0.45	0.50
ECU	20–200	0.171	0.139	0.120	0.083	0.050	**0.030**	0.031	0.087	0.159	0.330
	201–500	0.054	0.028	0.025	**0.010**	0.026	0.052	0.079	0.107	0.160	0.266
ED	20–200	0.085	0.041	0.027	**0.027** ^a^	0.062	0.124	0.176	0.253	0.379	0.719
	201–500	**0.011**	0.034	0.061	0.069	0.096	0.145	0.171	0.225	0.288	0.478
ECRB	20–200	0.226	0.146	0.064	**0.025**	0.119	0.222	0.335	0.486	0.664	0.931
	201–500	0.108	0.052	**0.013**	0.046	0.111	0.190	0.254	0.364	0.469	0.589
ECRL	20–200	0.213	0.177	0.147	0.118	0.088	0.062	**0.029**	0.040	0.099	0.175
	201–500	0.083	0.059	0.036	0.014	**0.008**	0.013	0.045	0.084	0.130	0.180
FCU	20–200	0.093	0.038	**0.022**	0.029	0.076	0.119	0.172	0.236	0.359	0.515
	201–500	**0.013**	0.018	0.042	0.071	0.101	0.130	0.160	0.194	0.274	0.640
FDSprox	20–200	**0.045**	0.062	0.186	0.294	0.468	0.748	0.911	1.367	2.173	26.652
	201–500	**0.020**	0.095	0.179	0.241	0.371	0.483	0.634	0.762	0.900	1.071
FDSdist	20–200	0.170	0.096	**0.044**	0.050	0.144	0.242	0.369	0.517	0.713	0.937
	201–500	0.045	**0.017**	0.054	0.105	0.159	0.226	0.304	0.362	0.479	0.613
FCR	20–200	0.084	0.043	**0.013**	0.042	0.073	0.117	0.202	0.291	0.409	0.616
	201–500	0.025	**0.005**	0.033	0.062	0.082	0.105	0.167	0.229	0.294	0.429

^a^ decision based on the fourth decimal (ath 0.15: 0.0272; ath 0.20: 0.0270).

The bold values indicate the smallest difference for each ROI.

**FIGURE 6:**
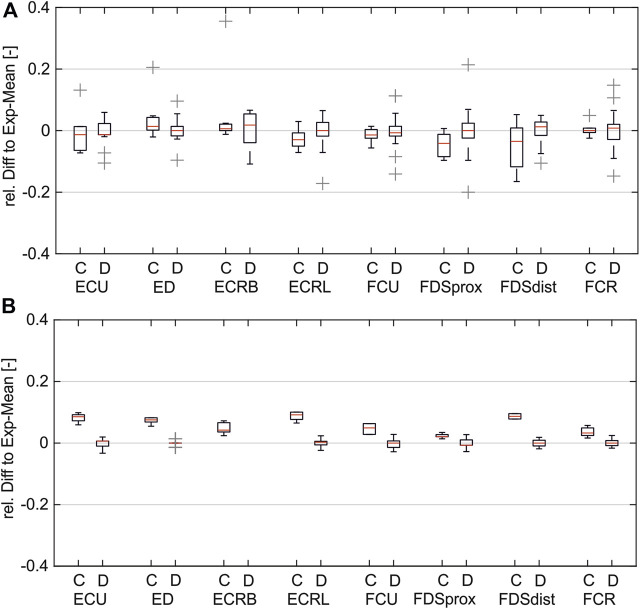
Comparison of simulated and inter-experimental distribution of relative differences: **(A)** for PW range 20–200 µs; **(B)** for PW range 201–500 µs. (C = difference between simulated data and experimental mean; D = difference between single experiments and mean).

Figure 6 shows the distribution of the relative differences between simulated PW to the experimental mean values (A) compared to the distribution of the inter-experimental differences (single experiments to experimental mean). The comparison shows that for both pulse width (PW) intervals, the remaining differences in the simulated values are in the same range as the inter-experimental differences for all muscles. It can be assumed that for a higher number of experiments, the experimental mean converges against the simulated curve.

**FIGURE 7:**
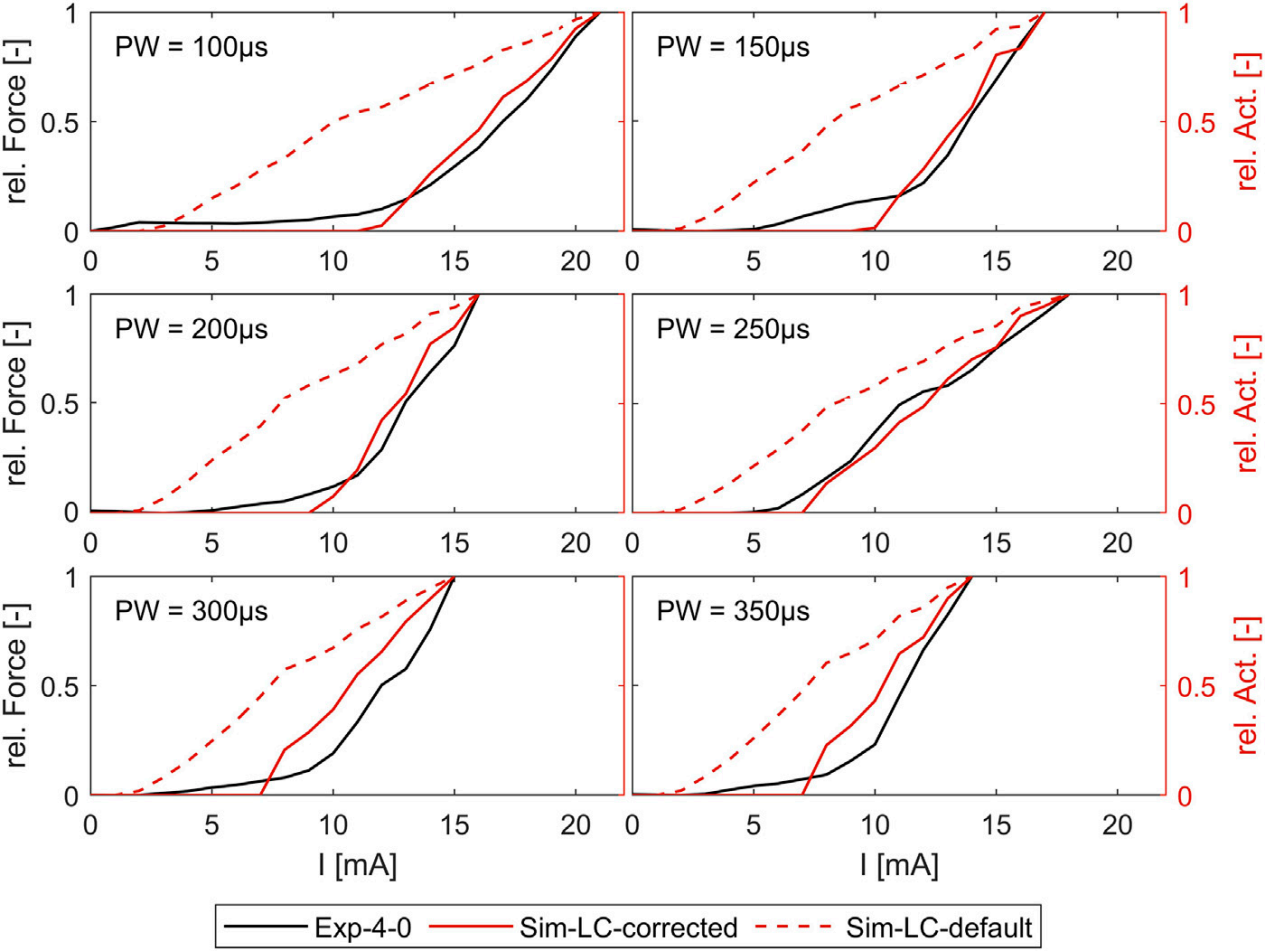
Comparison of simulated force-intensity curves (with and without, including the muscle-specific ath) with experimental curves for different PW. Example shown for the extensor carpi radialis muscle (ECRB).

Figure 7 shows the comparison of the simulated intensity-force curves with (corrected) and without (default) inclusion of the muscle-specific activation threshold (ath) with the experimentally measured intensity-force curves for different PWs for one muscle (ECRB) as an example. The experiment in this case was performed additionally and was not part of the determination of the activation thresholds. As can be seen, the corrected simulated curve fits well with the experimental curve for all PWs. Both curves show the characteristic onset of the sigmoidal progression of intensity-force curves. The characteristic late bending cannot be seen as the amplitude was not increased until the maximum muscle force was reached. The default simulated curve instead differs clearly from the experimental curve and does not show the characteristic sigmoidal progression.

## 4 Discussion

Despite the established use of FES for many years and extensive research, current applications at the forearm to stimulate hand movements are still limited to simple movements due to the difficulties of achieving selective muscle activations. Most research in this area has focused on experimentally determining motor points (MPs) using multichannel systems with electrode arrays and manual or semi-automatic search algorithms (Salchow et al., 2016a; Malesevic et al., 2012; Keller et al., 2007; Koutsou et al., 2016). However, the results and their impact are limited, mainly due to the small number of experiments (time-consuming, premature muscle fatigue). Simulations have been used successfully in the past to complement experimental research, but existing simulation models are not suitable for analyses of selective muscle activation.

This study used a new modelling approach to develop an individual forearm model that allows the analysis of FES at the level of individual muscle activation through simulations. Like existing models, our simulation model represents the “outer” anatomy by homogeneous tissue layers (bone, muscle, fat, and skin), as in, for example, Kuhn and Keller (2005), Loitz et al. (2015), RaviChandran et al. (2020b), and Gomez-Tames et al. (2013b). In contrast to these models, the shape of our tissue layers is neither fully simplified (as e.g. in Kuhn and Keller, 2005) nor fully anatomic (as e.g. in Gomez-Tames et al., 2013b). The shape of our model is anatomically based, meaning that an individual anatomy given by MRI data has been approximated with regular geometries—mostly ellipses. The advantage of this choice is that the resulting volumes and surfaces are still mathematically defined and, therefore, the model can be easily extended or modified. This is important:

• to allow the modeling of electrodes of any size and geometry (including electrode arrays in future applications) at any location on the skin;
• to allow future parameterization of the model, enabling its easy adaptation to different fat thicknesses or forearm lengths, for example.

As all changes to the electrode configuration are automatically updated via scripts and macros, the model can be easily integrated into optimization loops. Optimization loops can be used, for example, to find the best individual stimulation positions, which could then be integrated into an individual forearm sleeve.

In addition to the “outer” anatomy, our model also includes aspects of the “inner” anatomy; more precisely, it includes muscle-specific regions of interest (ROIs) that represent the muscle areas where the nerve entry point (NEP) and the innervation zone (IZ) of individual muscles are located. The nerve fibers were arranged in these various muscle-specific ROIs, which differ from existing models, which cover three main types of arrangement: single fiber or one group of fibers placed at a specific depth (e.g., Kuhn et al., 2010; Goffredo et al., 2014; RaviChandran et al., 2020b), uniform distribution of various fibers covering the whole muscle layer (e.g., Kuhn and Keller, 2007), and fibers arranged in one muscle-specific region (e.g., Gomez-Tames et al., 2013a; Loitz et al., 2015). In the latter case, this region was placed in the muscle layer based only on general anatomical information; in our model, the muscle-specific regions were placed based on the transfer of literature data regarding the position of NEPs to the underlying anatomy (MRI data). Due to the muscle-specific nerve fiber arrangement, our model enables the simulation of single muscle activations and, consequently, simulation-based analyses of selective muscle activation.

The experimental evaluation shows an overall good coincidence of the simulated and experimentally measured muscle activations. The small RSD of the different measurements indicate that the chosen reference system provides a robust method for transferring electrode positions between the digital twin and real forearm (Figure 4). The comparison of the simulated strength–duration (SD) curves with the measured curves shows that the simulations with the LC model are generally suitable for reproducing the characteristic course of the SD curves, unlike the simulations with the AF. The best fit is achieved when splitting the considered pulse width (PW) range in two intervals: 20–200 μs and 201–500 μs. For each muscle, different activation thresholds showed the best results. The definition of a global activation threshold would always lead to high differences in some muscles and is only used for the muscles not yet considered experimentally. Considering these limitations for low PWs up to 200 μs, the maximal mean relative difference between simulated and experimental curve is 0.045 and, for PWs higher than 200 μs, 0.013.

The main reason for the need to split the considered PW range in two intervals is that the measured amplitudes still decrease for high PWs. With increasing PW, the amplitude normally converges toward a minimum amplitude required to stimulate a muscle response—the rheobase (Baker et al., 1993). According to Baker et al. (1993), PW higher than 300 μs does not usually affect the stimulation amplitude. Therefore, we are currently working on evaluating a second existing person-specific model to understand whether this effect is person-specific or occurs more often and that perhaps the reporting studies have a bias, such as due to less precise measurement systems.

In addition to this experimental anomaly, there exist different simulation-based reasons that could explain the lack of the simulated curves to represent the measured curves over the full PW range.

• The selected nerve fiber orientation: all nerve fibers are modeled parallel to the ROI, which means parallel to the muscle course. This may not represent the actual fiber orientations with sufficient accuracy, as the terminal nerve branch splits after the NEP, and the individual fibers pull in all directions to reach all muscle fibers. It has been shown that the relative orientation of the nerve fibers to the electrode (if not rotationally symmetrical) influences activation (Loitz et al., 2015).
• The selected number of nerve fibers: the simulation is performed with a fixed number of 500 nerve fibers, which is probably higher than the real number (McComas, 1998). This number was chosen to minimize the influence of randomness.
• The selected nerve model type: the LC model is a very simplified model of a nerve fiber, assuming perfect insulation at the internodal sections. More detailed models consider the different ionic currents through membranes, such as the MRG model (McIntyre et al., 2002). These models could be integrated as well (e.g., Kuhn and Keller, 2005; RaviChandran et al., 2020b), but this increases simulation time significantly and is therefore a disadvantage when aiming for integration in optimization or AI methods.
• The selected parameters for the nerve model: changing individual model parameters influences the activation results and, consequently, also SD curves (McIntyre and Grill, 1998). The model parameters were chosen in accordance with exiting simulation studies in FES but may differ with the differing objectives of simulation models.

The first two of these simulation-based reasons may affect different muscles in different ways, and therefore might also affect the “global activation threshold” aspect mentioned above. However, an important question here is, “Is there one global activation threshold for all muscles?”. Measured SD curves for different muscles show differences (Baker et al., 1993), which indicates it is not. This is consistent with anatomical studies showing that fiber type distribution differs between muscles (Hwang et al., 2007). Based on this hypothesis, the definition of mean values as realized for the muscles not measured will lead to inappropriate muscle activations for these muscles. Therefore, we are currently working on individual measurements of these models to determine muscle-specific activation thresholds.

Considering the muscle-specific activation thresholds for the two PW intervals leads to promising results in predicting muscle activation independently of the stimulation intensity, which is determined by PW and amplitude. The simulated strength-intensity curves fit additional experimental data not used in prior determination of the activation thresholds and shows the characteristic force increase at the same amplitude. As muscle activation patterns are composed of single muscle activations, it can be assumed that the model is also suitable for simulating these and enabling further conclusions on stimulated movements or forces when several muscles are activated simultaneously, as is usual.

Overall, the approach presented holds significant promise of being suitable for designing digital twins of the forearm that can enable accurate and reliable prediction of real stimulated muscle activations. The next steps cover the two aspects mentioned above: experimental evaluation of a second individual model, which is already designed, and experimental evaluation of the remaining ROIs for the model presented. Furthermore, we aim to use the activation patterns simulated with the current model to predict the resulting movement or force. In a first step, each measured force could be related to a weighted sum of the activations of all muscles affecting this force location and direction (e.g., all extensors analyzed in these studies contribute to an extension in the wrist). In a second step, the model presented could be coupled with a biomechanical musculo-skeletal model to predict and visualize real movements. The current model is already suitable for integration into product development processes due to the automated model and simulation workflow modification: it can be integrated in optimization loops to find the best stimulation positions and design a patient-specific forearm sleeve for easy stimulation setup, or it can be used for developing optimized search algorithms for common electrode arrays with regular grids. In order to increase the benefits of this digital twin for product development, a future parameterization is planned. Such a parameterized digital twin will enable the prediction of muscle-specific activations by FES on the forearm for different forearm anatomies, which will particularly further improve the development of universal solutions using optimization or AI methods.

## Data Availability

The raw data supporting the conclusion of this article will be made available by the authors, without undue reservation.
